# Ginsenoside Rg1 improves anti-tumor efficacy of adoptive cell therapy by enhancing T cell effector functions

**DOI:** 10.1097/BS9.0000000000000165

**Published:** 2023-06-30

**Authors:** Yue Liu, Lingna An, Chengfei Yang, Xiaoqi Wang, Ruihao Huang, Xi Zhang

**Affiliations:** a Medical Center of Hematology, Xinqiao Hospital, State Key Laboratory of Trauma, Burn and Combined Injury, Army Medical University, Chongqing 400037, China; b Department of Urology, Xinqiao Hospital, Army Military Medical University, Chongqing 400037, China; c Jinfeng Laboratory, Chongqing 401329 China

**Keywords:** Anti-tumor efficacy, Adoptive cell therapy, CAR-T, Ginsenoside Rg1, Metabolic regulation

## Abstract

Adoptive cell therapy (ACT) has emerged with remarkable efficacies for tumor immunotherapy. Chimeric antigen receptor (CAR) T cell therapy, as one of most promising ACTs, has achieved prominent effects in treating malignant hematological tumors. However, the insufficient killing activity and limited persistence of T cells in the immunosuppressive tumor microenvironment limit the further application of ACTs for cancer patients. Many studies have focused on improving cytotoxicity and persistence of T cells to achieve improved therapeutic effects. In this study, we explored the potential function in ACT of ginsenoside Rg1, the main pharmacologically active component of ginseng. We introduced Rg1 during the in vitro activation and expansion phase of T cells, and found that Rg1 treatment upregulated two T cell activation markers, CD69 and CD25, while promoting T cell differentiation towards a mature state. Transcriptome sequencing revealed that Rg1 influenced T cell metabolic reprogramming by strengthening mitochondrial biosynthesis. When co-cultured with tumor cells, Rg1-treated T cells showed stronger cytotoxicity than untreated cells. Moreover, adding Rg1 to the culture endowed CAR-T cells with enhanced anti-tumor efficacy. This study suggests that ginsenoside Rg1 provides a potential approach for improving the anti-tumor efficacy of ACT by enhancing T cell effector functions.

## 1. INTRODUCTION

Adoptive cell therapy (ACT) is a fourth option for treating tumors, following surgery, chemotherapy, and radiotherapy. It has been classified into 2 categories: nonspecific and specific ACT. The former includes lymphokine-activated killer cell therapy, cytokine-induced killer cell therapy and dendritic cell therapy, all of which can improve the response and cytotoxicity of immune cells without targeting specific antigens. The latter includes chimeric antigen receptor T (CAR-T) cell therapy and T-cell receptor engineered T (TCR-T) cell therapy, which can target specific antigens and kill tumor cells.^1,2^ CAR-T cell immunotherapy has achieved remarkable success in recent years, especially in malignant hematological tumors.^3–5^ In recurrent/refractory B-cell acute lymphoblastic leukemia (R/R B-ALL), the complete remission rate (CR) achieved with CAR-T cells can reach 90%, and a series of CAR-T cell therapeutic products has been approved in America and China.^6^ CAR-T has been gradually regarded as the most promising cancer therapy. However, the effectiveness is greatly reduced in solid tumors.^7,8^ The immunosuppressive tumor microenvironment (TME) causes 2 urgent and severe problems: insufficient killing activity and limited persistence of CAR-T cells. Hence, there is a pressing demand to further optimize CAR-T and other ACTs to improve efficiency, especially in solid tumors.

The principal in vitro CAR-T cell manufacturing process includes the following steps: (1) obtaining and isolating T cells from patients or donors, (2) enriching and modifying T cells with CAR structure transduction, and (3) drastically expanding CAR-T cells. Recently, studies have focused on changing the in vitro manufacturing process to improve anti-tumor activity.^9,10^ Strategies for greater cytotoxicity and prolonging persistence are 2 main optimization approaches. For example, knockout of several immune inhibitory regulators expressed on the T cell surface (PD-1, LAG-3, TIM-3, and CTLA-4) and coexpression of immune activators, interleukins and chemokine receptors have been implemented in CAR-T cells, and corresponding clinical trials are ongoing.^11–16^ Additionally, several conventional drugs have been reported to have novel applications in CAR-T cell therapy, functioning by regulating metabolism and differentiation to result in a redirection from an exhausted to a memory T-cell state, including decitabine, ibrutinib, metformin, and sulforaphane.^17–20^ Based on these studies, it has been demonstrated that the quality and characteristics of T cells from patients or donors critically affects the efficiency of CAR-T cells and other ACT products. Hence, exploring more suitable drugs or bioactive molecules is a promising approach for improving CAR-T cell therapy. These improvements would help to increase the efficiency and application of ACT products.

**Figure 1.**
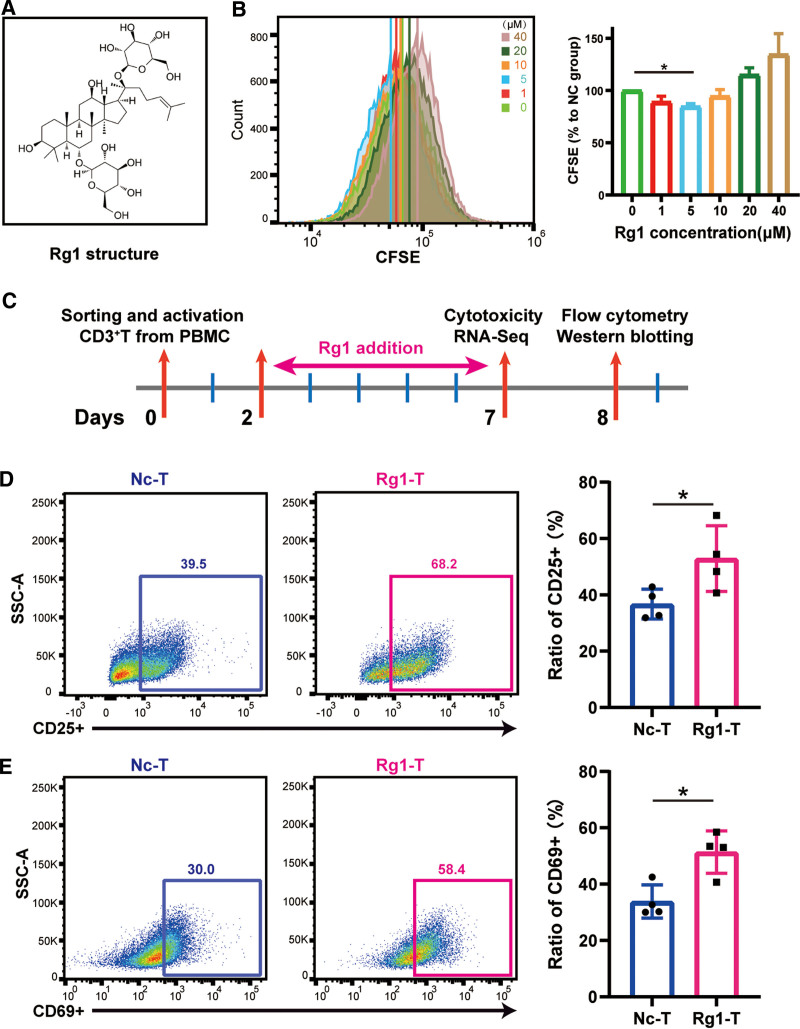
Rg1 enhances T cell activation and proliferation. (A) The structure of ginsenoside Rg1. (B) Dose-dependent effect of Rg1 in T cell culture indicated by a CFSE labeling assay. (C) Flowchart of the T cell activation and proliferation process and functional tests. D&E Flow cytometry plots and histograms of CD25 and CD69 expression in the negative control group and the Rg1-treated group. CFSE = carboxyfluorescein succinimidyl ester, NC = negative control group, PBMC = peripheral blood mononuclear cell. *P < .05, **P < .01, and ***P < .001 compared to negative control.

*Panax ginseng* C. A. Meyer (ginseng) is a valuable Chinese medicine, which has been used to treatment diverse diseases for more than 2000 years. It has been verified to boost the immune system, which has been regarded as its most significant traditional use.^21–24^ The main pharmacologically active component of ginseng is ginsenoside and it has been confirmed as having anti-inflammatory, anti-oxidation and anti-tumor functions, as well as improving immunity.^22,25,26^ More than 100 ginsenosides have been found and extracted.^27^ Notably, ginsenoside Rg1 is one of the main active components (**Fig. 1A**), and its pharmacological activities and underlying mechanisms have been thoroughly explored. 1. Anti-tumor activity: When co-cultured with the chronic myeloid leukemia cell line K562, Rg1 was able to induce tumor cell cycle arrest in the S phase and inhibit cell proliferation.^28^ 2. Antioxidative activity: Rg1 increased the survival rate of SH-SY5Y cells injured by hydrogen peroxide by reducing the amount of released lactate dehydrogenase (LDH) and increasing superoxide dismutase activity. Rg1 effectively suppressed caspase-3 immunoreactivity and contributed to heat shock protein 70 gene expression in a dose-dependent manner.^29^ 3. Immunoregulatory activity: A number of studies have shown that Rg1 improves immunity by modulating multiple immune cells.^30,31^ Lee et al found that Rg1 increased the proportion of T helper cells and promoted IL2 gene expression in murine splenocytes.^24^ In peripheral blood mononuclear cell (PBMC) derived dendritic cells, Rg1 upregulated the expression of the maturation marker CD83 and human leukocyte antigen-antigen D related isotype (HLA-DR), which suggested that Rg1 promoted the phenotypic maturation of human dendritic cells.^32^ Considering the potential effect on immunoregulatory activity, we planned to explore the regulating effect of ginsenoside Rg1 on ACTs. We introduced Rg1 during the in vitro activation and expansion phase of T cells isolated from human PBMCs. Two markers of T cell activation, CD69 and CD25, were upregulated and the maturation process of T cells was promoted by Rg1, which resulted in enhanced cytotoxicity. Transcriptome sequencing analysis revealed that Rg1 influenced the T cell metabolic reprogramming by strengthening mitochondrial biosynthesis. Moreover, when included in the CAR-T cells manufacturing process, Rg1 improved the anti-tumor efficacy in vitro, which suggested that ginsenoside Rg1 is a potential immunopotentiator for CAR-T cell therapy and other ACT products.

## 2. MATERIALS AND METHODS

### 2.1. Cell source and cell culture

Nalm6 cells with a firefly luciferase and GFP expression cassette (Nalm6-Luc) were cultured in RPMI-1640 (Biological Industries, Kibbutz Beit Haemek, Israel) supplemented with 10% FBS (Biological Industries), 100 U/mL penicillin and 0.1 mg/mL streptomycin (Beyotime, Shanghai, China). Human peripheral blood was obtained from healthy donors (n = at least 4) and was handled according to ethical, moral, and safety requirements. PBMCs were isolated with Ficoll-Paque Plus (Cytiva, Washington, DC, USA) via density gradient centrifugation. Then, CD3^+^ T cells were purified and stimulated with anti-CD3/CD28 beads (ThermoFisher, Waltham, MA, USA) (1:1 cell/bead ratio) in X-VIVO15 medium (Lonza, Basel, Switzerland) supplemented with 200 U/mL IL2 (MCE, Monmouth Junction, NJ, USA). The medium was changed every 2 days and the cell density was maintained at 10^6^ cells/mL. Rg1 (5 μM) (purity over 98.0%) (MCE) was added after 48 hours of stimulation and then supplied in fresh medium.

### 2.2. CD19 CAR-T cell production

A CD19 CAR cassette was designed based on the Kymirah product. The extracellular binding region was derived from the single-chain variable fragment (scFv) of an anti-CD19 antibody (FMC63); the extracellular spacer and transmembrane domain were derived from the CD8 protein; the co-stimulatory domain 41-BB was derived from CD137; and the intracellular signaling domain was derived from TCR/CD3z. The CAR structure was packaged into a lentivirus with an enhanced GFP cassette as an indicator. After 48 hours of culture, T cells were transfected with the CAR lentivirus at a multiplicity of infection of 10. The medium containing the lentivirus and anti-CD3/CD28 beads was removed 24 hours following transduction. The medium was changed every 2 days, and the cell density was maintained at 10^6^ cells/mL for other assays.

### 2.3. Cell proliferation assay

T cell and CAR-T cell proliferation was detected with the CFSE Cell Proliferation Assay and Tracking Kit (Beyotime). A total of 1 × 10^6^ cells were incubated with 1 mL carboxyfluorescein succinimidyl ester (CFSE) regent at 37°C for 10 minutes. Then, 10 mL of culture medium was added and gently mixed, followed by centrifugation at 300×g for 5 minutes. Then, 5 mL medium were added, and the cells were re-centrifuged. The cells were cultured with Rg1 at different concentrations (0, 1, 5, 10, 20, and 40 μM) and proliferation was detected by flow cytometry.

### 2.4. Flow cytometry assay

T/CAR-T cells were centrifuged at 300×g for 5 minutes and resuspended in 100 μL PBS with 2% FBS. The T/CAR-T cells were stained at 4°C for 30 minutes with the following surface marker-specific antibodies: APC/Cy7-anti-CD25 (BD Pharmingen, San Diego, CA, USA), APC-anti-CD69 (BioLegend, San Diego, CA, USA), PE-anti-CD4 (BD Pharmingen), PE/Cy7-anti-CD8 (BioLegend), PE-anti-CD45RO (BD Pharmingen), APC-anti-CD62L (BD Pharmingen), PE-anti-PD-1 (BioLegend), PE/Cy7-anti-CTLA-4 (BioLegend), APC-anti-TIM-3 (BioLegend), and APC anti-human CD127 (BioLegend). The cells were then washed twice and resuspended in 400 μL PBS with 2% FBS for flow cytometry.

### 2.5. Transcriptome sequencing

Negative control T cells (2 × 10^6^) and Rg1-treated T cells (2 × 10^6^) were washed, collected, and digested with TRIzol™ reagent (Sigma, St. Louis, MO, USA). Total RNA was extracted using chloroform, isopropyl alcohol, 75% ethanol, and diethylpyrocarbonate-treated water, following the manufacturer’s protocol. Transcript library construction and sequencing were performed by LC-bio (Hangzhou, China). Raw reads were then filtered to acquire clean reads. The mRNA levels of transcripts and genes were assessed according to the fragments per kilobase of transcript per million mapped reads (FPKM). Based on the FPKM values, different expression analysis was performed to compare the negative control T cell and Rg1-treated T cell groups.

### 2.6. Western blot

Cells were collected and lysed with NP40 Lysis Buffer (Beyotime) with 1 mM Phenylmethanesulfonyl fluoride (Beyotime). Each quantity of 10^6^ cells was lysed with 50 μL NP40 buffer. The cell lysates were collected by centrifugation at 13,000 rpm at 4°C for 10 minutes. Protein loading buffer (Beyotime) was added, and the samples boiled at 95°C for 10 minutes. The isolated protein was subjected to SDS-PAGE on 4% to 20% polyacrylamide gels (ACE Biotechnology, Nanjing, China) and transferred to NC membranes (Millipore, St. Louis, MO, USA). The membranes were incubated with a primary antibody and secondary antibody. The primary antibodies included anti-Glut1, anti-LDHA, anti-NRF1, anti-NRF2, anti-GZMB, anti-Actin (Beyotime), anti-SIRT1 (Abclonal, Wuhan, China), anti-HK2, anti-CPT1α, anti-PGC1, anti-MTCO2, anti-TFAM, anti-GZMA, anti-GZMK, anti-PRF1, anti-PD-1, anti-TIM-3, anti-CTLA-4, anti-NDUFB8, anti-SDHB, anti-MTCO1, anti-UQCRC2, and anti-ATP5A (Abcam, Cambridge, UK). The secondary antibodies included HRP-labeled goat anti-rabbit and HRP-labeled goat anti-mouse antibodies (Beyotime). Finally, the proteins were detected by West Femto Maximum Sensitivity Substrate (ThermoFisher).

### 2.7. Mitochondria detection assay

The mitochondrial mass of T cells was detected by MitoTracker Red CMXRos (Beyotime). A total of 1 × 10^6^ cells were centrifuged at 1000×g for 5 minutes and resuspended in 100 μL of preheated working buffer. The cells were incubated for approximately 30 minutes at 37°C, followed by centrifugation at 1000×g for 5 minutes. They were then resuspended in fresh culture medium and evaluated by flow cytometry.

### 2.8. Cytotoxicity assay

The cytotoxicity of T/CAR-T cells was evaluated with a luciferase-based assay. Approximately, 1 × 10^4^ Nalm6 cells expressing luciferase were added to the wells of a white 96-well plate, and T/CAR-T cells were added for co-incubation at different ratios and different times. The viability of the Nalm6 cells was detected with the Bio-Lite Luciferase Assay System (Vazyme, Nanjing, China). Killing activity was quantified as follows: lysis (%) = (no T/CAR-T cell groups) − (T/CAR-T cell co-culture groups)/(no T/CAR-T cell groups) × 100%.

### 2.9. ATP detection assay

The ATP levels in cells in the negative control group and the Rg1-treated group were quantitatively detected using the Enhanced ATP Assay Kit (Beyotime) according to the manufacturer’s instructions, using samples of 1 × 10^6^ cells. Then, the cellular ATP content was measured with a multimode reader.

### 2.10. Xenograft mouse model assay

For the in vivo study to evaluate CAR-T cell function, we established an intravenously injected Nalm6 ALL mouse model using female 6-week-old NPG mice (Beijing Vitalstar Biotechnology, Beijing, China). The mice received an injection of 200 μL RPMI-1640 containing 1 × 10^6^ Nalm6 cells on day 0, CAR-T Rg1-CAR-T cells were resuspended in X-VIVO15 and injected into mice through the tail vein on day 7. Tumor burdens were monitored and analyzed by BURKER In-Vivo XtremeII Platform on day 14.

## 3. RESULTS

### 3.1. Ginsenoside Rg1 promotes T cell activation and influences phenotypic characteristics

Given that ginsenoside Rg1 has been found to promote T cell activation in murine splenocytes, we first evaluated the influence of Rg1 on T cell proliferation during the in vitro activation phase. CD3^+^ T cells were isolated from PBMCs of healthy donors, and after a 48 hours activation with anti-CD3/CD28 beads, the cells were exposed to a series of Rg1 concentrations. A CFSE labeling assay indicated that a 72-hour Rg1 treatment had dual effects on CD3^+^ T cells. When the concentration of Rg1 was under 5 μM, the proliferation rate increased, while when it was over 5 μM, the viability decreased (**Fig. 1B**). These phenotypes suggested that the regulatory effect of Rg1was related to concentration. Based on these results, we chose 5 μM Rg1 for subsequent assays to maximally activate CD3^+^ T cells (**Fig. 1C**). In response to T cell priming and activation, two surface markers, CD69 and CD25, were promptly upregulated and transduced activation signals from the extracellular TCR domain into the organelles and the cell nucleus, and these markers also faithfully reflected the TCR signaling strength. Flow cytometry demonstrated that Rg1 addition significantly increased the CD69^+^ T and CD25^+^ T cell proportions and expression levels (**Fig. 1D and E**), which demonstrated that Rg1 could promote T cell activation.

**Figure 2.**
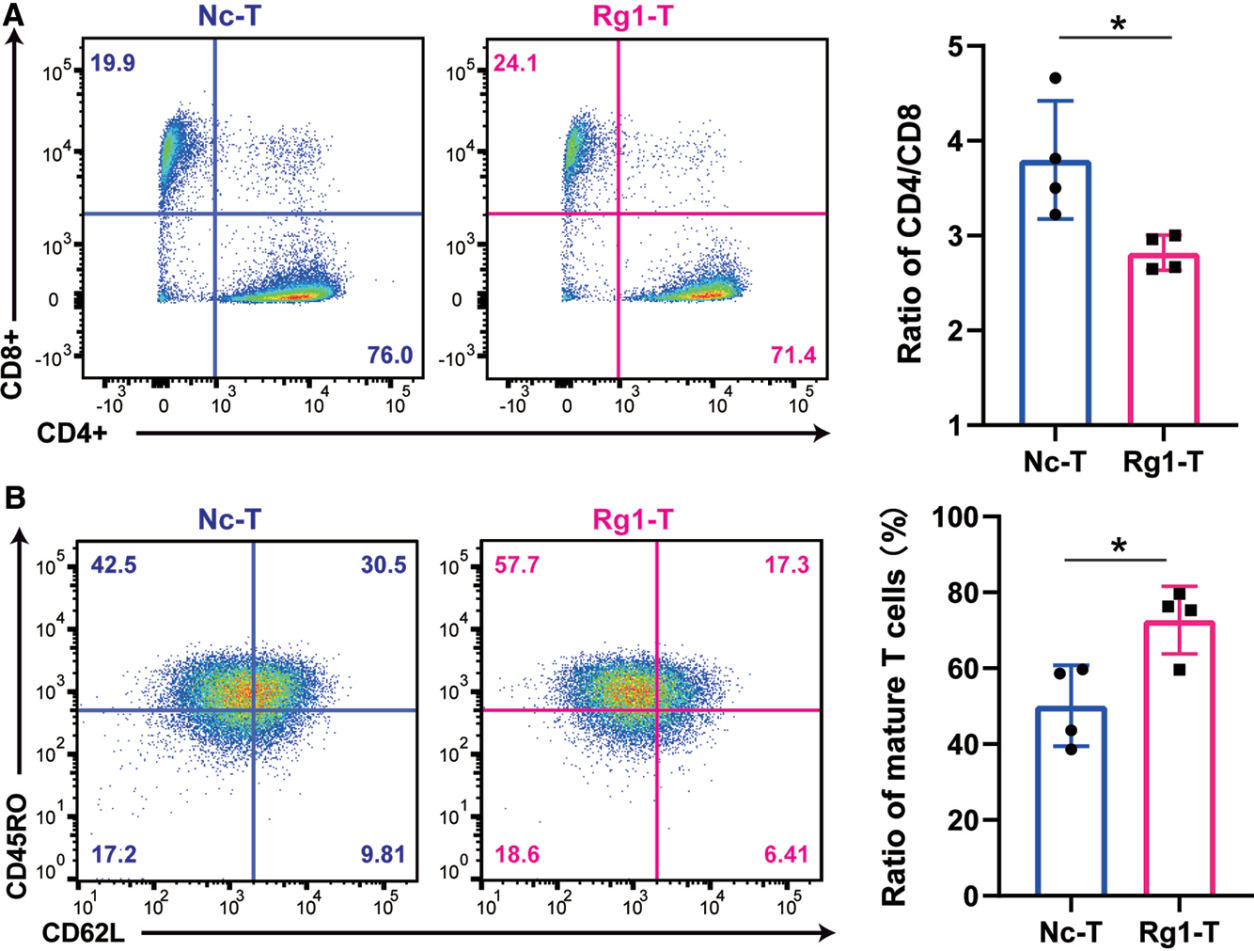
Rg1 influences T cell phenotypic characteristics. (A) Flow cytometry plots and histograms of CD4 and CD8 expression in the negative control group and the Rg1-treated group. (B) Flow cytometry plots and histograms of CD45RO and CD62L expression in the negative control group and the Rg1-treated group. *P < .05, **P < .01, and ***P < .001 compared to negative control.

CD4^+^ and CD8^+^ T cells are the 2 main subgroups of CD3^+^ T cells, and the ratio of CD4/CD8 is not constant among individuals. Traditional adoptive T cell therapy is based on the CD3^+^ T cells.^33^ Recently, several studies have demonstrated that the CD4/CD8 ratio is connected with the therapeutic efficacy in clinical trials.^34,35^ Therefore, the CD4/CD8 ratio in the ACT products should be considered, and we assessed the ratio after Rg1 exposure. The CD4/CD8 ratio decreased after 6 days of Rg1 exposure, implying that CD8^+^ T cells were more strongly activated and proliferated more quickly in response to Rg1, than CD4^+^ T cells (**Fig. 2A**). During the T cell activation and proliferation phase, naive T cells gradually acquired a mature T cell phenotype, CD62L^low^ and CD45RO^high^, which was also regarded as the phenotype of effector T cells. This population is the main group responsible for cytotoxicity. By flow cytometry, we found that the addition of Rg1 had significantly increased the size of the mature T cell subset at day 8, increasing from 50.1% to 72.7% (**Fig. 2B**). These results suggested that ginsenoside Rg1 could promote T cell activation and drive the differentiation toward a mature T cell subset.

### 3.2. Ginsenoside Rg1 treatment induces T cell metabolic reprogramming

**Figure 3.**
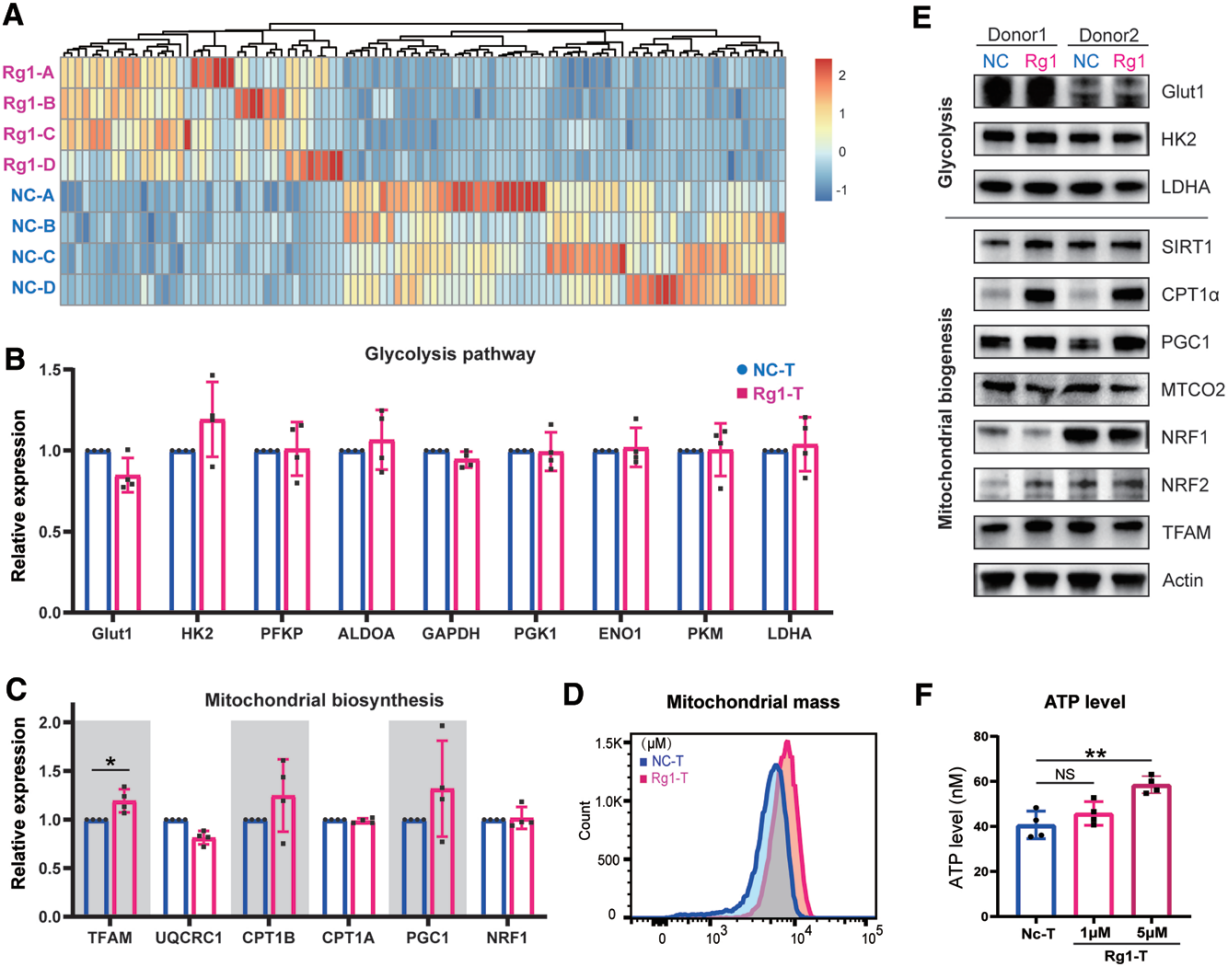
Transcriptional signatures and metabolic characteristics of T cells after Rg1 treatment. (A) Hierarchical clustering of transcriptome sequencing analysis results identified differentially expressed genes between the negative control group and the Rg1-treated group (n = 4). (B) Normalized enrichment scores for several typical glycolysis pathway-related genes. (C) Normalized enrichment scores for several typical mitochondrial biosynthesis-related genes. (D) The mitochondrial mass detected with flow cytometry analysis using MitoTracker. (E) Western blot analysis of glycolysis and mitochondrial biosynthesis-related genes. (F) Detection of the cellular ATP content among different groups. *P < .05, **P < .01, and ***P < .001 compared to negative control.

To further explore the molecular mechanisms underlying the alternations in activation and maturation on ginsenoside Rg1 treatment, we performed transcriptome sequencing of Rg1-treated and negative control T cells from 4 healthy donors on day 7 after anti-CD3/CD28 stimulation. There was homogeneity to a large extent but some heterogeneity among the different donors (**Fig. 3A**). It has been reported that ginsenosides are able to regulate metabolic processes, including ATP production, glucose absorption and utilization, and mitochondrial biosynthesis.^36^ Moreover, glycolysis in the cytoplasm and oxidative phosphorylation (OXPHOS) in the mitochondria are 2 main metabolic pathways in different T cell subsets.^37^ The field has gradually reached the consensus that metabolic reprogramming between glycolysis and OXPHOS occurs during T cell activation, which is critical for the clinical effect of adoptive T cell therapy.^38^ Several studies have demonstrated that improving glycolysis and OXPHOS in T cell therapy could enhance anti-tumor activity and persistence.^39,40^ Based on this, we focused on the metabolic pathways in the transcriptome. First, we found that there were no significant changes in the expression levels of several key glycolysis-related genes (**Fig. 3B**). For OXPHOS, we selected several typical genes involved in mitochondrial biosynthesis (**Fig. 3C**). Notably, mitochondrial transcription factor A (TFAM), PPAR gamma coactivator 1 (PGC1), and carnitine palmitoyltransferase 1β (CPT1B) were upregulated at the transcriptional level. We then performed western blot assay to detect the expression levels of proteins involved in glycolysis and mitochondrial biosynthesis. We found that the level of Glut1, HK2 and LDHA were similar between the negative control group and the Rg1-treated group. The protein expression of sirtuin 1 (SIRT1), carnitine palmitoyltransferase 1 α (CPT1α), PGC1, and NFE2-like bZIP transcription factor 2 (NRF2) was significantly upregulated (**Fig. 3E**). Moreover, we also supplemented 5 marker genes for mitochondrial complexes I–V, but the expression showed no notable change (Supplemental Figure S1E, http://links.lww.com/BS/A61). In parallel, these upregulated genes needed for mitochondrial biosynthesis induced a greater mitochondrial mass, higher 2-NBDG uptake and reactive oxygen species (ROS) production with Rg1 exposure (**Fig. 3D**, Supplemental Figure S1B, http://links.lww.com/BS/A61). The cellular ATP level was significantly increased by 5 μM Rg1 treatment (**Fig. 3F**), while the LDH activity showed no noticeable change (Supplemental Figure S1C, http://links.lww.com/BS/A61). Overall, these results revealed that Rg1 addition induced greater mitochondrial biosynthesis and ATP production in T cells.

### 3.3. Ginsenoside Rg1 enhances the cytotoxicity of T cells

**Figure 4.**
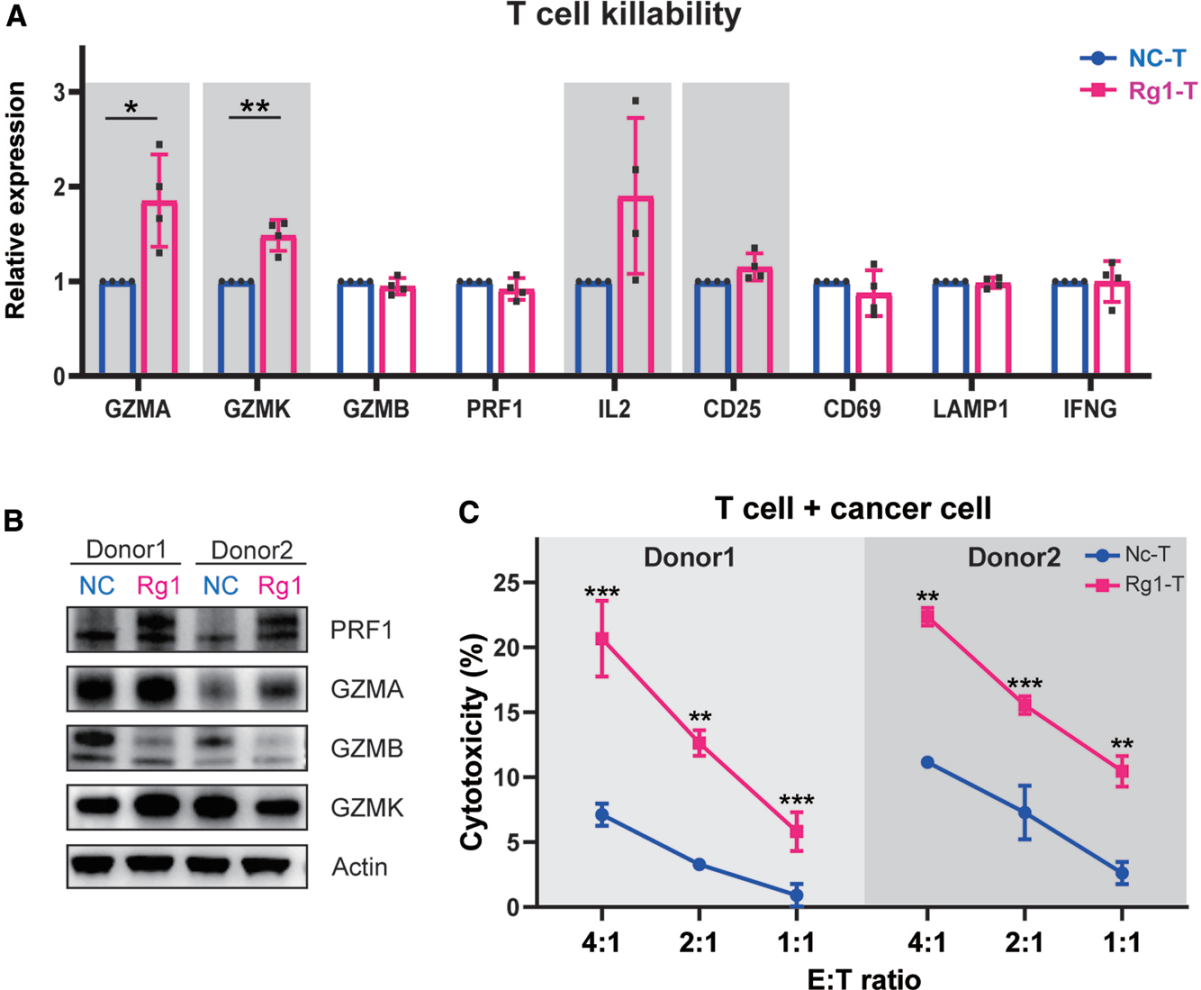
Rg1-pretreated T cells exhibit enhanced anti-tumor activity. (A) Normalized enrichment scores for several typical cytotoxicity-related genes. (B) Western blot analysis of PRF1, GZMA, GZMB, and GZMK. (C) Cytotoxicity analysis of Rg1-pretreated T cells and negative control T cells co-cultured with Nalm6-Luc cells at effector: target cell ratios of 4:1, 2:1, and 1:1. *P < .05, **P < .01, and ***P < .001 compared to negative control.

Rg1 treatment promoted T cell activation and maturation by enhancing mitochondrial biosynthesis, which is regarded as a critical factor for T cell differentiation and function. We then speculated that Rg1 might regulate the effector function of T cells. According to the transcriptome sequencing results, we selected key genes responsible for T cell cytotoxicity, including members of the granzyme family (GZMA, GZMB, GZMK), perforin 1 (PRF1), IL2, CD25, CD69, LAMP1 and interferon gamma (IFNG), comparing them between the Rg1-treated group and the negative control group. GZMA and GZMK were significantly upregulated, and the expression of IL2 and CD25 was slightly increased, while other targets showed no significant change at the transcriptional level (**Fig. 4A**). Correspondingly, the protein levels of PRF1 and GZMA were clearly upregulated, as shown by western blot (**Fig. 4B**), which suggested that the effector function of T cells was enhanced by Rg1 treatment. The discrepancies between the transcriptional and translational levels of these proteins were not difficult to understand because of post-translational modification. Then, we compared the anti-tumor effects of Rg1-pretreated T cells and normal T cells. Co-cultured with Nalm6-Luc cells at different effector: target cell ratios (4:1, 2:1, and 1:1) for 6 hours showed that the cytotoxicity of T cells treated with Rg1 was obviously strengthened (**Fig. 4C**). This result suggested that Rg1 pretreatment could improve the expression of cytotoxic factors and enhance the cytotoxicity of T cells to tumor cells. Collectively, these results implied that Rg1 could be regarded as a synergist for ACTs.

### 3.4. Ginsenoside Rg1 enhances the anti-tumor efficacy of CAR-T cells

**Figure 5.**
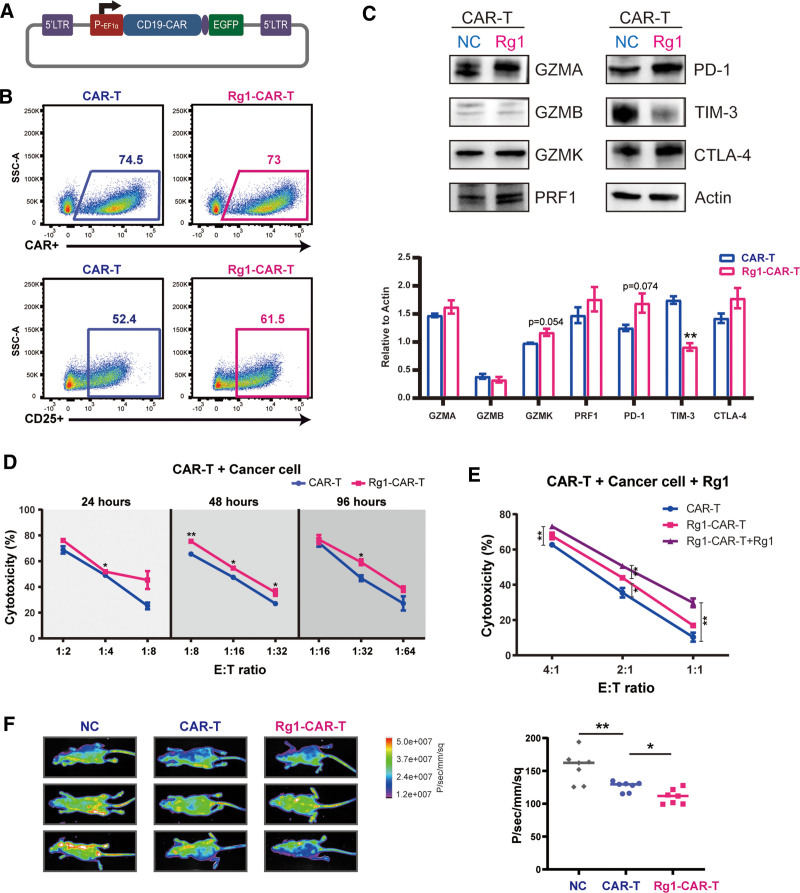
CAR-T cells improved by Rg1 treatment. (A) The structure of the CAR expression cassette. (B) Flow cytometry analysis of CAR and CD25 expression in the negative control CAR-T group and the Rg1-treated CAR-T group. (C) Western blot analysis of cytotoxicity-related proteins and exhaustion-related proteins. (D) Analysis of the cytotoxicity Rg1-pretreated CAR-T cells and the negative control CAR-T cells co-cultured Nalm6-Luc cells at different effector: target cell ratios and different incubation times. (E) Analysis of the cytotoxicity of Rg1-treated CAR-T cells consistently treated with Rg1. (F) The tumor burden in 3 groups of Nalm6-bearing mice was assessed by bioluminescent imaging. CAR-T = chimeric antigen receptor T. *P < .05, **P < .01, and ***P < .001 compared to negative control.

Based on the above observation, it was evident that Rg1 was able to enhance T cell anti-tumor activity. We therefore treated CAR-T cells with Rg1 to explore whether Rg1 could improve the CAR-T-based therapeutic efficacy. We selected a typical CD19 CAR structure, which was approved by the FDA for use in R/R B-ALL patients, for CAR-T cell construction (**Fig. 5A**). Rg1 addition synchronized with the CAR construct lentiviral transduction. First, we found that Rg1 addition did not influence the CAR expression level (**Fig. 5B**), which indicated that the promotion of activation and maturation characteristics by Rg1 was not dependent on CAR expression. The proportion of CD25^+^ T cells was increased, just as negative control T cells (**Fig. 5B**). Considering this upregulated expression of CD25, we also investigated Treg cells; the Treg cell ratio was not significantly increased (Supplemental Figure S1D, http://links.lww.com/BS/A61). Western blot analysis showed that GZMA and PRF1 were upregulated in CAR-T cells treated with Rg1. Furthermore, it has been reported that the promotion of activation and maturation in T cells generally indicates a tendency towards exhausted characteristics. Therefore, we investigated several typical exhaustion markers (**Fig. 5C**, Supplemental Figure S1A, http://links.lww.com/BS/A61). PD-1 was significantly upregulated, the CTLA-4 level was slightly increased, and TIM-3 was downregulated, revealing the complexity of the regulatory effects of Rg1 on different exhaustion markers. Further, a co-culture assay demonstrated that Rg1-pretreatment enhanced the cytolytic activity of CD19 CAR-T cells at different effector: target cell ratios and incubation times (**Fig. 5D**). Moreover, we found that Rg1 addition could further improve the cytotoxic effects of CAR-T cells when co-cultured with Nalm6-Luc cells (**Fig. 5E**). We subsequently evaluated the CAR-T cell functionality *i*n vivo using xenograft mouse models. As expected, Nalm6-bearing mice with Rg1 pretreated CAR-T cells demonstrated slightly lower tumor burdens compared with the traditional CAR-T group; this needs further optimization for improving efficacy (**Fig. 5F**).

Collectively, these data indicated that the enhanced cytotoxicity of T cells induced by Rg1 treatment could be used to improve CAR-T therapeutic efficacy and Rg1 might be combined with traditional ACT products to achieve this.

## 4. DISCUSSION

The application of ACT products, especially those used in CAR-T cell therapy, has become one of the most promising clinical therapy methods for tumors. Multiple approved products and clinical trials have revealed that CAR-T is highly efficient against B-cell malignant hematological tumors, in which the CR reaches almost 90%.^6,41^ However, the success rate of CAR-T cells against other types of leukemia or solid tumors is much lower than that against B-ALL, mainly because of the insufficient cytotoxicity and persistence.^42^ Several studies have been devoted to improving the cytotoxicity and persistence of CAR-T cells and ACT products.^10,38,43,44^ Genetic engineering and manipulation have been widely adopted to produce a superior T cell phenotype. For example, CD19 CAR-T cells with PD-1 integration showed a greater ability to eradicate tumor cells in xenograft models and clinical trials.^11,13^ Knockout of the receptor of adenosine, a key immunosuppressive metabolite in TME, increased the capacity to inhibit solid tumor growth and development.^45,46^ On the other hand, several traditional drugs have, surprisingly, been found to regulate CAR-T cell differentiation, which were potential synergists for ACT products. Metformin, known for reducing glucose levels and treating type II diabetes, has been confirmed to reprogram T cell differentiation and maintain memory T cell characteristics, resulting in superior persistence.^19^ These findings indicated a direction for CAR-T optimization to achieve better cytotoxicity and longer persistence.

We have focused on ginseng, a traditional Chinese medicine. The active components, ginsenosides, have been used to enhance immunity, eradicate inflammation and limit cancer.^30^ Based on the fact that ginsenoside was able to activate T cells and promote cytokine secretion, in this study, we explored the function of Rg1 during the in vitro expansion phase of T cells isolated from human PBMCs. We first found that Rg1 addition enhanced T cell activation and proliferation induced by anti-CD3/CD28 beads. Two activation markers expressed on the cell surface, CD69 and CD25, were significantly upregulated (**Fig. 1**). Moreover, the proportions of different subsets of T cells showed that Rg1 promoted the T cell maturation process, with a larger mature population indicating more rapid killing of tumor cells (**Fig. 2**). For some tumors, the cytotoxicity of T cells is universally defective because of the long-term suppression by TME. Hence, Rg1 might partly supplement the activity of patient-derived T cells. Subsequently, to explore the underlying regulatory mechanism of Rg1, we performed transcriptomic analyses, and found that mitochondrial biosynthesis, which has been regarded as an indicator of ACT performance *i*n vivo and in vitro, was significantly increased (**Fig. 3**).

The notable changes observed in the activation and maturation phenotypes of T cells prompted us to explore whether Rg1 treatment affects the T cell effector function. PRF1, GZMA, and GZMK, 3 key effector molecules, were shown to be upregulated, and Rg1-pretreated T cells showed a stronger tumor lytic function at different effector: target cell ratios (**Fig. 4**). Furthermore, we introduced Rg1 during the CAR-T cell manufacturing phase. Consistent with the result for T cells, the activation and cytotoxicity of CAR-T cells were significantly enhanced (**Fig. 5**). Notably, the addition of Rg1 to the co-culture containing CAR-T cells and tumor cells further improved the lytic function and Rg1 pretreated CAR-T cells demonstrated a better anti-tumor effect than the traditional CAR-T group in vivo using xenograft mouse models. Overall, we verified that Rg1 could improve the anti-tumor efficacy of both T cells and CAR-T cells. However, existing side effects indicate the need for further optimization before Rg1 application in ACTs. One barrier is that Rg1 promoted T cell maturation, but the memory cell proportion was downregulated, which was closely related to T cell persistence.^47^ Another reason is that PD-1 was upregulated by Rg1 treatment, which allowed easier induction of T cell exhaustion. We plan to overcome these barriers through combination with PD-1 gene knockout.^48^ Additionally, more than 100 varieties of ginsenosides need further evaluation to select the best example.

**Figure 6.**
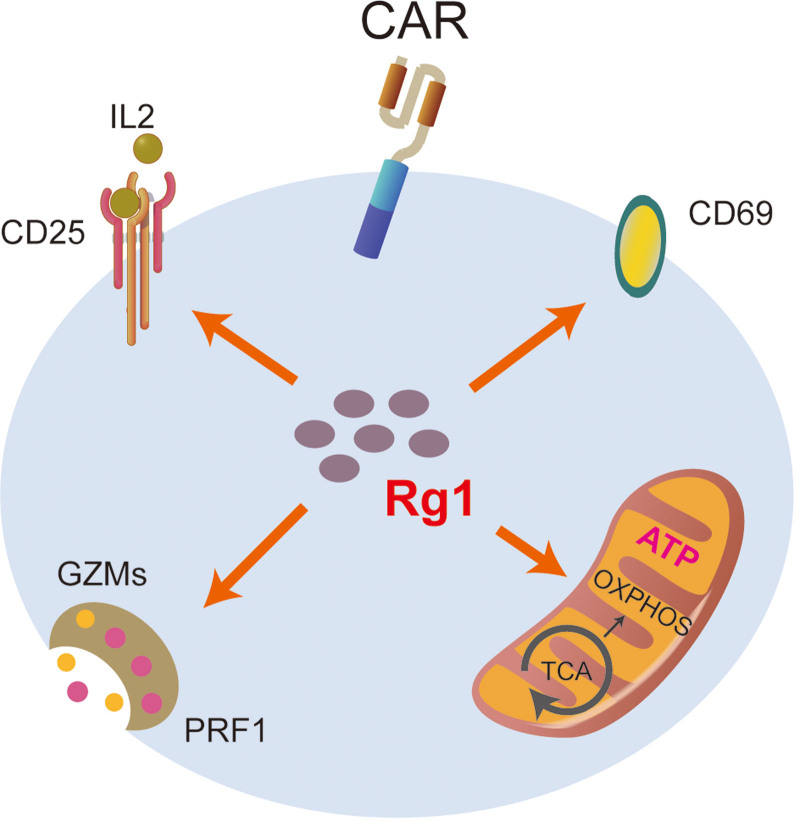
Model for illustrating the underlying activation-related mechanism by which Rg1 affects T cells. CAR = chimeric antigen receptor, PRF1 = perforin 1.

In summary, our study revealed that ginsenoside Rg1 could enhance the activation and proliferation of T cells. Mitochondrial biosynthesis and effector functions were significantly improved (**Fig. 6**). Moreover, the effect was consistent in CAR-T cells. These results suggest that ginsenoside Rg1 has the potential to become a synergist for ACT products and promote their application.

## ACKNOWLEDGMENTS

This work was supported by the National Natural Science Foundation of China (No. 82020108004 and 81873424), the Natural Science Foundation of Chongqing, China (No. CSTB2022NSCQ-MSX1287), Special Funding for the Frontiers of Military Medical Basics (No. 2018YQYLY002), Key Technical Innovation Projects in Clinical Fields of Xinqiao Hospital (No. 2018JSLC0020), and the Young Doctor Talent Incubation Program of Xinqiao Hospital (No. 2022YQB016).

## AUTHOR CONTRIBUTIONS

Y.L. and L.A.: conducting the experiments, writing, original draft preparation; C.Y.: performing the data analysis, conducting the in vitro and in vivo experiments; R.H. and X.W.: performing data analysis, conceptualization; X.Z.: data curation, conceptualization, writing, reviewing, and editing.

## Supplementary Material


