# Supplementary figures and images for: Ginsenoside Rg1 improves anti-tumor efficacy of adoptive cell therapy by enhancing T cell effector functions
Source: Blood Sci. 2023 Jun 30;5(3):170–9. doi: 10.1097/BS9.0000000000000165 (PMC10400057; doi:10.1097/BS9.0000000000000165)

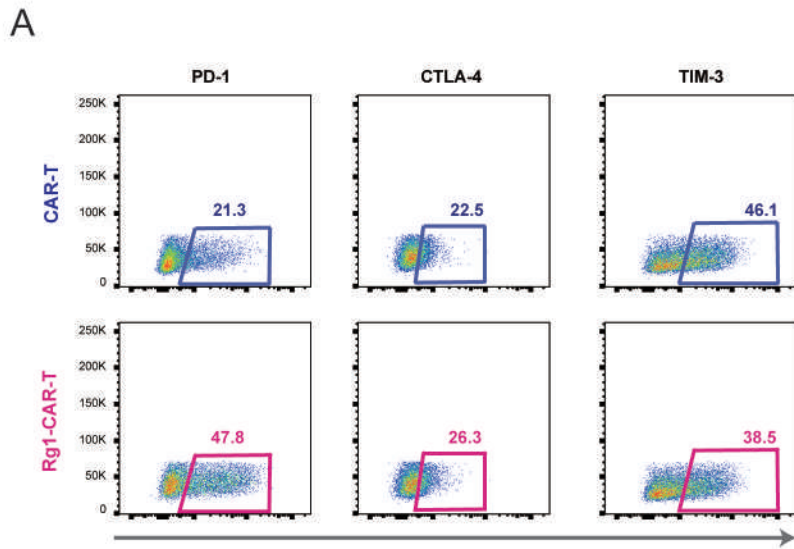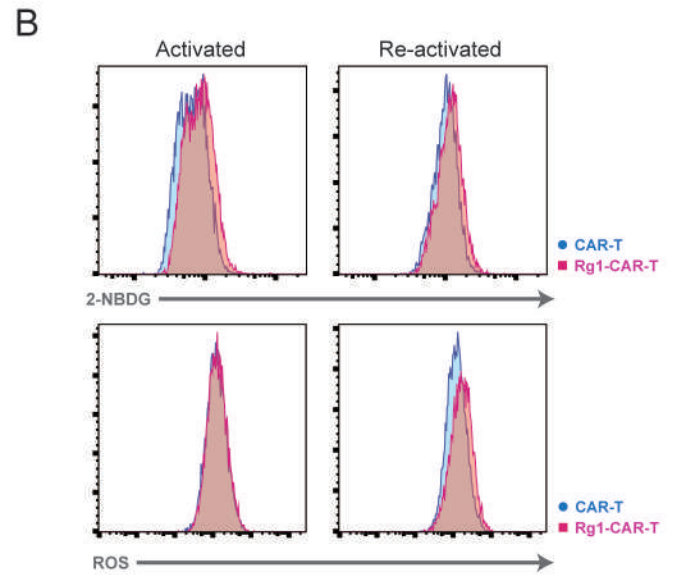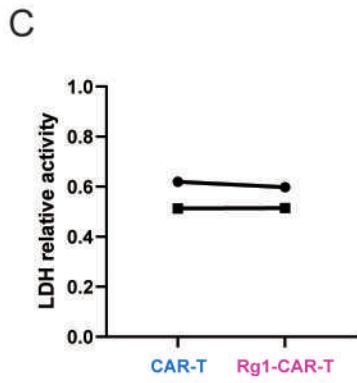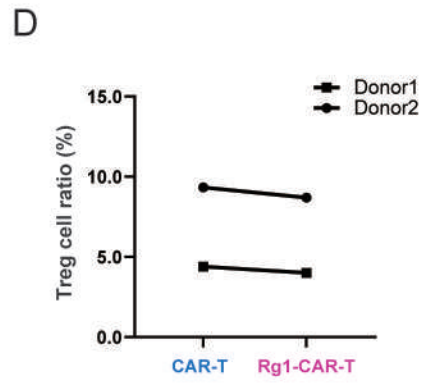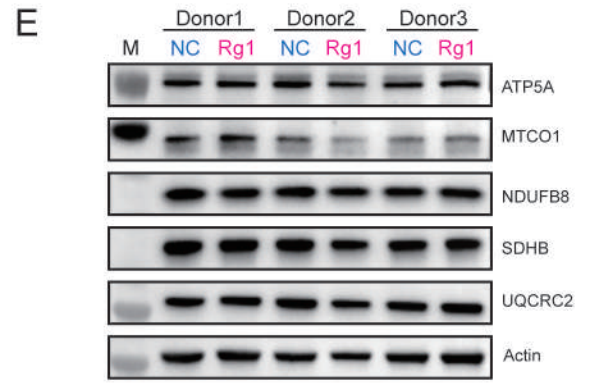

Supplement: Supplementary file 1 [file bs9-5-170-s001.pdf]
